# Supplementary material for: Behavior change due to COVID-19 among dental academics—The theory of planned behavior: Stresses, worries, training, and pandemic severity
Source: PLoS One. 2020 Sep 29;15(9):e0239961. doi: 10.1371/journal.pone.0239961 (PMC7523990; doi:10.1371/journal.pone.0239961)
Supplement: S1 Appendix — (PDF) [file pone.0239961.s001.pdf]

Appendix 1

Table 1: Countries participating in the study and number of participants

Country	Number of invited academics	Number of participants	Percentage to all participants	Response rate
Bosnia	100	55	3.0	55.0
Brazil	1350	104	5.6	7.7
Denmark	71	10	0.5	14.1
Egypt	310	85	4.6	27.4
France	630	30	1.6	4.8
Germany	1400	149	8.0	10.6
India	1662	208	11.2	12.5
Indonesia	200	129	6.9	64.5
Iran	700	268	14.4	38.3
Italy	527	51	2.7	9.7
Japan	280	89	4.8	31.8
Jordan	100	67	3.6	67.0
Kenya	60	4	0.2	6.7
Korea	220	31	1.7	14.1
KSA	90	51	2.7	56.7
Libya	103	14	0.8	13.6
Myanmar	100	13	0.7	13.0
Nigeria	86	54	2.9	62.8
Norway	70	7	0.4	10.0
Palestine	53	13	0.7	24.5

Peru	150	13	0.7	8.7
Serbia	400	9	0.5	2.3
Syria	150	12	0.6	8.0
Thailand	470	34	1.8	7.2
UAE	77	12	0.6	15.6
UK	150	56	3.0	37.3
USA	6820	248	13.3	3.6
Yemen	200	46	2.5	23.0
Total	16,529	1,862	100	11.3
