# Supplementary material for: Behavior change due to COVID-19 among dental academics—The theory of planned behavior: Stresses, worries, training, and pandemic severity
Source: PLoS One. 2020 Sep 29;15(9):e0239961. doi: 10.1371/journal.pone.0239961 (PMC7523990; doi:10.1371/journal.pone.0239961)
Supplement: S2 Appendix — (DOCX) [file pone.0239961.s002.docx]

**Appendix 2**

**English version**

Dental academics' stresses at the time of the COVID-19 outbreak

This questionnaire is targeting academics in dental institutions, please respond to it only if you are working in a dental academic institution. Your responses are confidential and cannot be traced to you. They will not be shared with anyone and only the research team will have access to them.

1. Please rate how distressed you are because of the COVID-19 outbreak on a scale of not at all, rarely, sometimes and often:

1. I think about it when I don't mean to.
2. I avoid letting myself get upset when I think about it or get reminded about it.
3. I try to remove it from memory.
4. I have trouble falling asleep or staying asleep because of pictures or thoughts about it that come to my mind.
5. I have waves of strong feelings about it.
6. I have dreams about it.
7. I stay away from reminders about it.
8. I feel as if it haven't happened or is unreal.
9. I try not to talk about it.
10. Pictures about it pop into my mind.
11. Other things make me think about it.
12. I am aware that I have a lot of feelings about it, but I don't deal with them.
13. I try not to think about it.
14. Any reminder brings feelings about it.
15. My feelings about it are kind of numb.

2. On a scale from 1 (not worried at all) to 10 (extremely worried), please rate each of the following:

1. Catching COVID-19 infection from a colleague at work
2. Catching COVID-19 infection from a patient during treatment
3. Catching COVID-19 infection from a student
4. Catching COVID-19 infection from a source not related to work
5. Important others/ loved ones getting infected with COVID-19 because of me
6. Important others/ loved ones getting infected with COVID-19 because of another source
7. Patients getting infected with COVID-19
8. Finishing open courses satisfactorily because of the COVID-19 outbreak
9. Teaching students required material because of the COVID-19 outbreak
10. Supporting students psychologically in difficult times of the COVID-19 outbreak
11. Managing extra load required to provide online learning because of the COVID-19 outbreak
12. Finishing required reports/ assignments before important deadlines because of the COVID-19 outbreak
13. Restricted mobility from one place/ city to another in my country because of the COVID-19 outbreak
14. Restricted mobility from and to my country because of the COVID-19 outbreak
15. Restricted mobility affecting my sports and social activities because of the COVID-19 outbreak
16. Missing scientific events important to my career (conferences, presentations, meetings..) Because of the COVID-19 outbreak

3. On a scale from 1 (strongly disagree) to 10 (strongly agree), indicate how much you agree with the following:

1. After the COVID-19 outbreak, I changed my life habits
2. After the COVID-19 outbreak, I wash my hands more frequently
3. After the COVID-19 outbreak, I avoid crowded places
4. After the COVID-19 outbreak, I cancel social events
5. After the COVID-19 outbreak, I cancelled travel plans

4. Gender

- Male
- Female
- No response

5. Age

- 25-35
- 36-45
- 46-55
- 56-65
- 66+

6. Home/living arrangements

- Live alone
- Live with parents
- Live with partner/spouse
- Live in shared accommodation
- Other

7. Country

8. Highest academic degree

- BDS (or equivalent)
- M.Sc (or equivalent)
- Phd (or equivalent)

9. Coordinates courses

- No
- Yes

10. Deals with patients

- No
- Yes

11. Did the faculty staff receive any training on dealing with public health emergencies?

- Yes
- No

12. Do you have any administrative roles?

- Yes
- No

**Portuguese version**

Nível de estresse no corpo docente do curso de Odontologia em tempos de pandemia pelo COVID-19

Este questionário é direcionado a acadêmicos em instituições odontológicas. Por favor, responda a ele apenas se você estiver trabalhando em uma instituição acadêmica odontológica. Suas respostas são confidenciais e não podem ser rastreadas até você. Eles não serão compartilhados com ninguém e apenas a equipe de pesquisa terá acesso a eles.

## Informe o quão estressado(a) o(a) senhor(a) se sente em relação à atual pandemia dizendo nunca, raramente, às vezes ou frequentemente para cada uma das frases abaixo.

1. Penso nisso quando não gostaria de pensar.
2. Tento não ficar chateado(a) quando penso nisso ou sou lembrado(a) disso.
3. Tento apagar isso da minha memória.
4. Tenho dificuldades para dormir ou para me manter dormindo devido às imagens e pensamentos que vem à minha cabeça.
5. Ao pensar nisso sinto um turbilhão de emoções.
6. Sonho com isso.
7. Eu me afasto de coisas que me fazem lembrar disso.
8. Sinto como se isso não tivesse acontecido ou não fosse real.
9. Tento não falar disso.
10. Imagens sobre isso pipocam na minha cabeça.
11. Outras coisas aleatórias me fazem lembrar disso.
12. Tenho consciência de que tenho muitos sentimentos em relação a isso, mas não lido com eles.
13. Tento não pensar sobre isso.
14. Qualquer coisa que me lembre disso me afeta.
15. Eu me sinto insensível, desconectado, quando ouço falar sobre a pandemia.

## Em uma escala de 1 a 10, informe como se sente com as afirmações a seguir. A nota 1 significa “não me preocupa em nada” e a nota 10 significa “me preocupo muito”.

1. Pegar COVID-19 de um colega de trabalho.
2. Pegar COVID-19 de um paciente durante o tratamento.
3. Pegar COVID-19 de um aluno.
4. Pegar COVID-19 de outra fonte não relacionada ao trabalho.
5. Pessoas importantes para mim, que eu amo, pegarem COVID-19 por causa de mim.
6. Pessoas importantes para mim, que eu amo, pegarem COVID-19 por outras fontes.
7. Pacientes pegarem COVID-19.
8. Oferecer um atendimento adequado aos meus pacientes em meio à pandemia.
9. Concluir atividades curriculares de forma satisfatória em meio à pandemia.
10. Ensinar aos meus alunos os conteúdos programados em meio à pandemia.
11. Dar apoio emocional aos meus alunos nesses tempos difíceis.
12. Dar conta da carga extra de trabalho para oferecer preparar aulas em plataformas online.
13. Concluir relatórios e tarefas dentro dos prazos estipulados em meio à pandemia.
14. Mobilidade restrita devido às medidas de saúde pública em meu país.
15. Trânsito restrito para sair ou entrar em meu país devido à pandemia.
16. Perder eventos importantes para minha profissão como congressos, reuniões e apresentações devido à pandemia.
17. Em uma escala de 1 (discordo totalmente) a 10 (concordo plenamente), indique o quanto o(a) senhor(a) concorda com as afirmações a seguir.
18. Em virtude da pandemia do COVID-19 eu mudei meus hábitos de vida.
19. Em virtude da pandemia do COVID-19 eu lavo as mãos mais frequentemente.
20. Em virtude da pandemia do COVID-19 eu evito lugares com aglomerações de pessoas.
21. Em virtude da pandemia do COVID-19 eu cancelei eventos sociais.
22. Em virtude da pandemia do COVID-19 eu cancelei planos de viagem.
23. Gênero

- Masculino
- Feminino
- Sem resposta

1. Idade

- 25-35
- 36-45
- 46-55
- 56-65
- 66+

1. Como você vive

- Vivo sozinho
- Vivo com os meus pais
- Vivo com um parceiro / cônjuge
- Vivo em alojamento compartilhado
- Outros

1. País
2. Maior grau acadêmico

- Especialização (ou equivalente)
- Mestrado (ou equivalente)
- Doutorado (ou equivalente)

1. Você coordena cursos

- Sim
- Não

1. Você vê pacientes

- Sim
- Não

1. Os professores receberam algum treinamento para lidar com emergências de saúde pública?

- Sim
- Não

1. Você tem alguma função administrativa?

- Sim
- Não

**Farsi version**

**
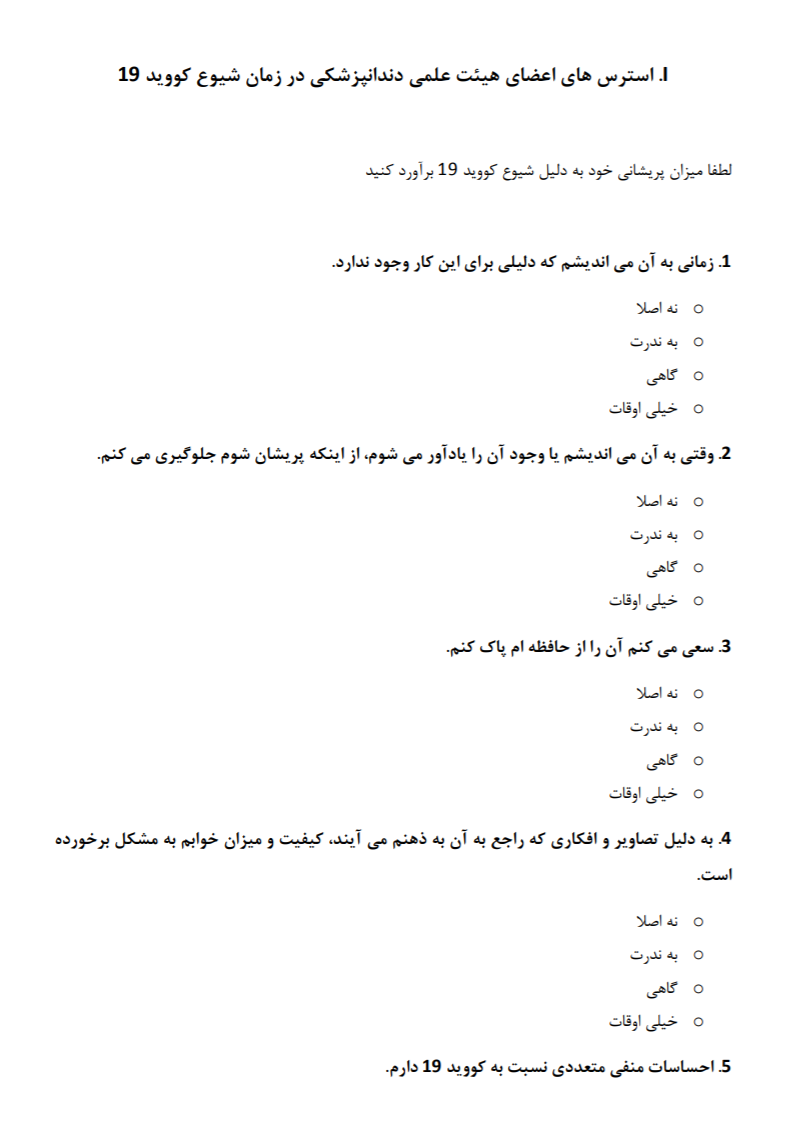

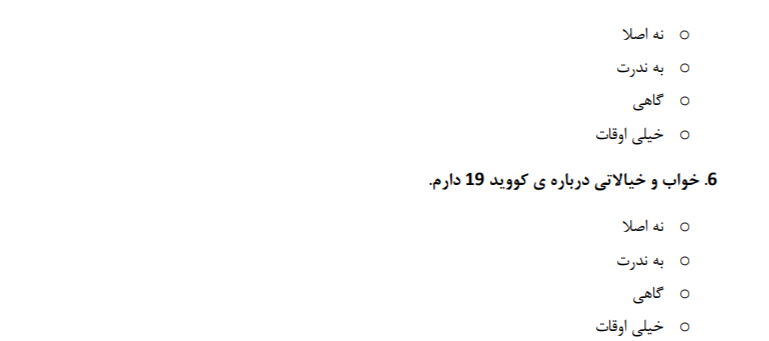
**

**
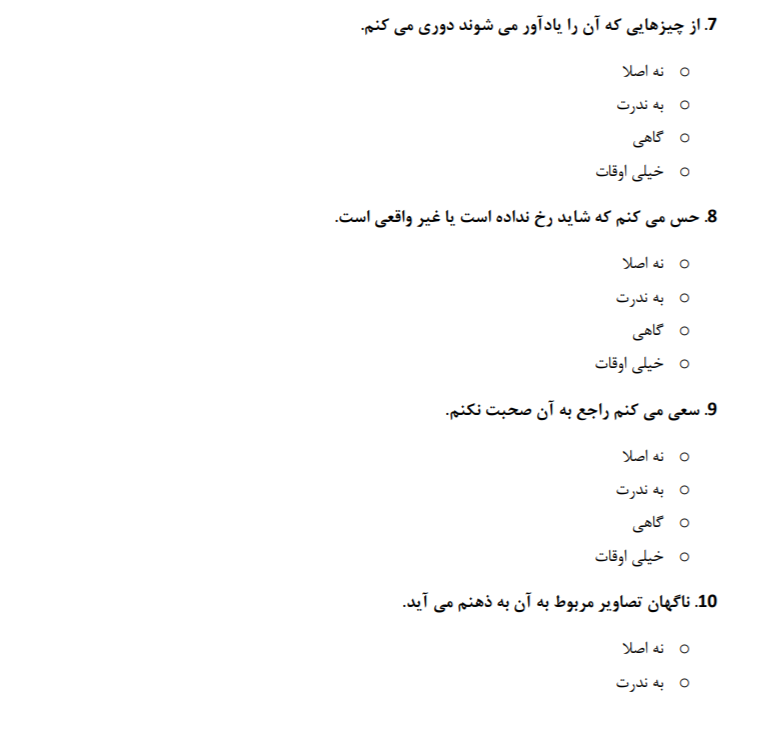

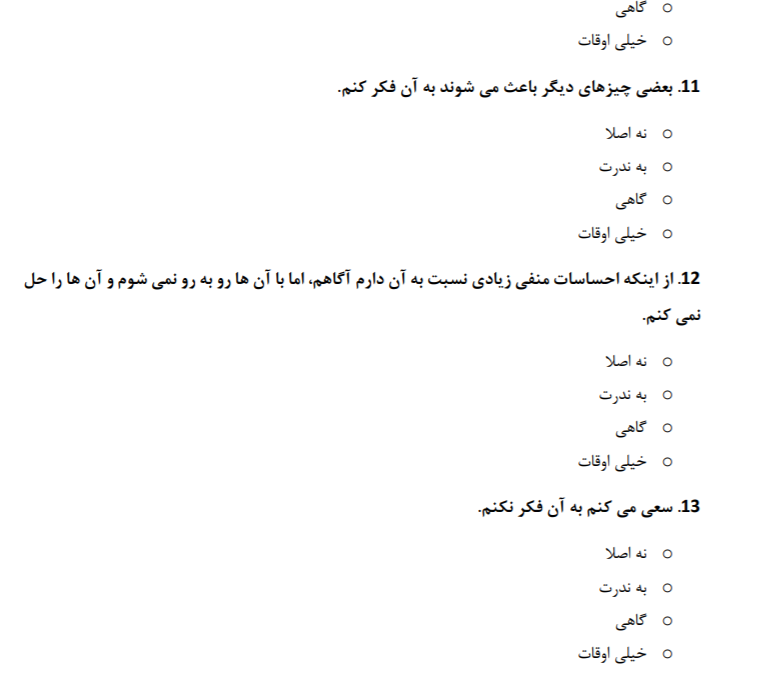
**

**
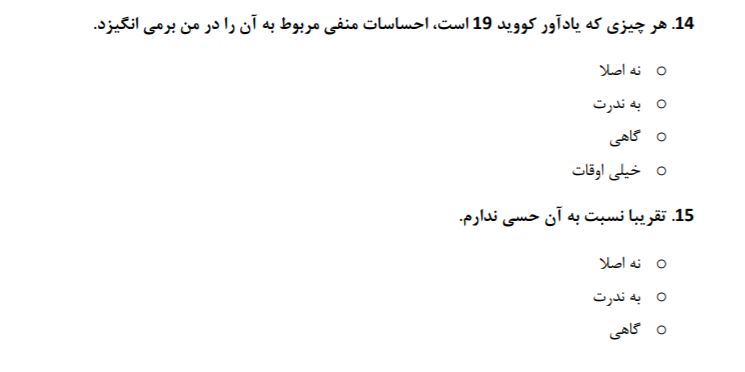
**

**
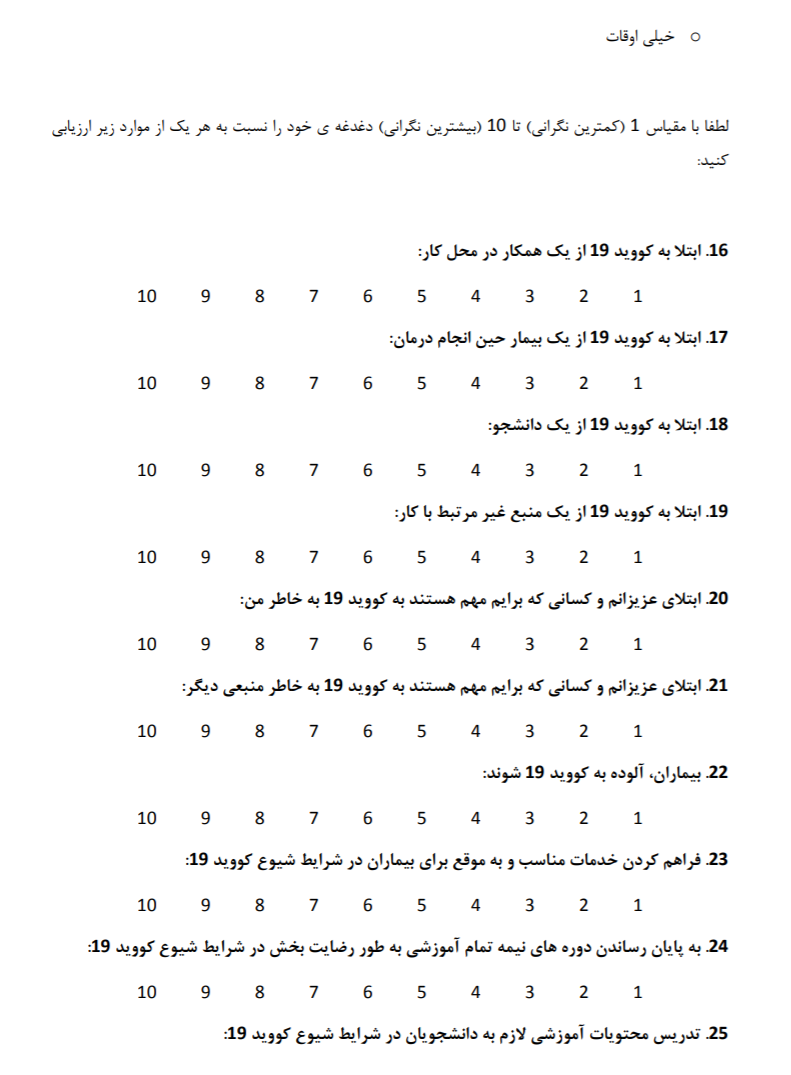
**

**
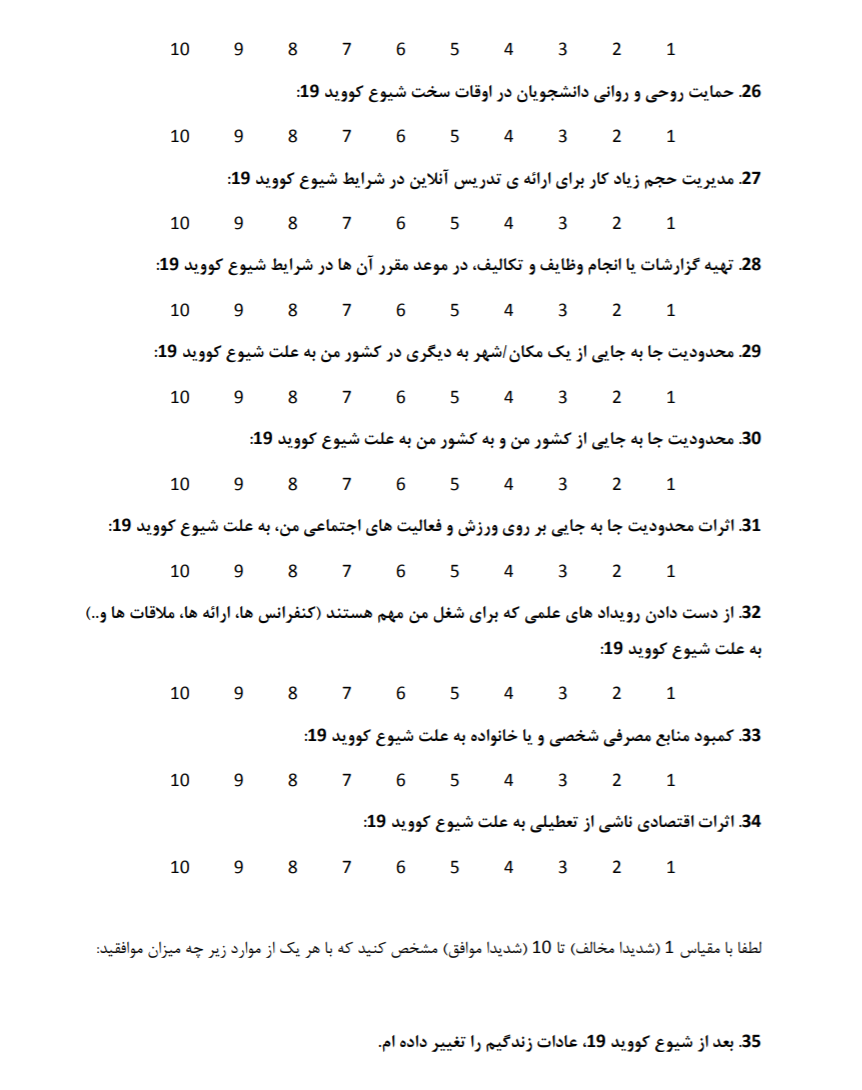
**

**
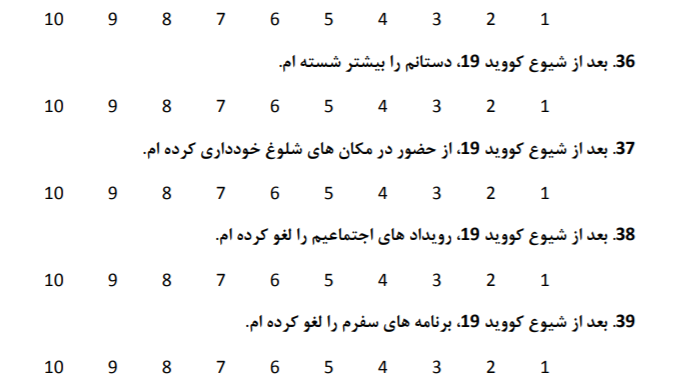
**
