# Supplementary material for: Behavior change due to COVID-19 among dental academics—The theory of planned behavior: Stresses, worries, training, and pandemic severity
Source: PLoS One. 2020 Sep 29;15(9):e0239961. doi: 10.1371/journal.pone.0239961 (PMC7523990; doi:10.1371/journal.pone.0239961)
Supplement: Attachment — Submitted filename: PONE-D-20-20341_reviewer (1) Comments.pdf [file pone.0239961.s004.pdf]

PLOS ONE

Behavior change due to COVID-19 among dental academics - The theory of planned behavior: stresses, worries, training, and pandemic severity

--Manuscript Draft--

Manuscript Number:	PONE-D-20-20341
Article Type:	Research Article
Full Title:	Behavior change due to COVID-19 among dental academics - The theory of planned behavior: stresses, worries, training, and pandemic severity
Short Title:	Dental academics' COVID-19 behavior
Corresponding Author:	Maha El Tantawi<br>Alexandria University Faculty of Dentistry<br>Alexandria, Alexandria EGYPT
Keywords:	Dental faculty; COVID-19; psychological stresses; fear; training; surveys and questionnaires; multilevel analysis
Abstract:	Purpose<br>COVID-19 pandemic led to major life changes. We assessed the psychological impact of COVID-19 on dental academics globally and on changes in their behaviors.<br><br>Method<br>We invited dental academics to complete a cross-sectional, online survey from March to May 2020. The survey was based on the Theory of Planned Behavior (TPB). The survey assessed stress levels (using the Impact of Event Scale), fears, and worries because of COVID-19 extracted by Principal Component Analysis (PCA) (attitudes in the TPB), participants' training to manage public health emergencies (control in the TPB). and personal and professional backgrounds. We used multilevel regression models to assess the association between frequent handwashing and avoidance of crowded places (outcome variables) and explanatory variables; stresses, fears, training, and country-level COVID-19 deaths to cases ratio representing fatality rate (norms in the TPB).<br><br>Results<br>1862 academics from 28 countries participated in the survey (response rate= 11.3%). Of those, 53.4% were female and 32.9% were <46 years old. Severe stress was identified in 9.9%. PCA extracted three main factors: fear of infection, worries because of professional responsibilities, and because of restricted mobility. These factors showed a significant dose-dependent association with stress levels and were also significantly associated with more frequent handwashing (B= 0.56, 0.33, and 0.34) and avoiding crowded places (B= 0.55, 0.30, and 0.28). Low fatality rates were significantly associated with more handwashing (B= -2.82) and avoiding crowded places (B= -6.61). Training was not significantly associated with behavior change (B= -0.01 and -0.11).<br><br>Conclusions<br>COVID-19 had a considerable psychologic impact on dental academics. There was a direct, dose-dependent relationship between change in behaviors and worries and fears but no association with training. More change in behaviors was associated with lower COVID-19 fatality rates. Fears and stresses were associated with greater adoption of preventive measures against the pandemic.
Order of Authors:	Nour Ammar
	Nourhan M. Aly
	Morenike O. Folayan
	Yousef Khader
	Jorma Virtanen
	Ola B. Al-Bateynah

Powered by Editorial Manager® and ProduXion Manager® from Aries Systems Corporation

	Simin Z. Mohebbi
	Sameh Attia
	Hans-Peter Howaldt
	Sebastian Boettger
	Diah A. Maharani
	Anton Rahardjo
	Imran Khan
	Marwa Madi
	Maher Rashwan
	Verica Pavlic
	Smiljka Cicmil
	Youn-Hee Choi
	Easter Joury
	Jorge L. Castillo
	Kanako Noritake
	Anas Shamala
	Gabriella Galluccio
	Antonella Polimeni
	Prathip Phantumvanit
	Davide Mancino
	Jin-Bom Kim
	Maha M. Abdelsalam
	Arheiam AlAwami
	Mai A. Dama
	Myat Nyan
	Iyad Hussein
	Mohamed M. AlKeshan
	Ana P. Vukovic
	Alfredo Iandolo
	Arthur M. Kemoli
	Maha El Tantawi

**Additional Information:**	
**Question**	**Response**
**Financial Disclosure**<br><br>Enter a financial disclosure statement that describes the sources of funding for the work included in this submission. Review the submission guidelines for detailed requirements. View published research articles from *PLOS ONE* for specific	The authors received no specific funding for this work.

examples.<br><br>This statement is required for submission and **will appear in the published article** if the submission is accepted. Please make sure it is accurate.<br><br>**Unfunded studies**<br>Enter: *The author(s) received no specific funding for this work.*<br><br>**Funded studies**<br>Enter a statement with the following details:<br>• Initials of the authors who received each award<br>• Grant numbers awarded to each author<br>• The full name of each funder<br>• URL of each funder website<br>• Did the sponsors or funders play any role in the study design, data collection and analysis, decision to publish, or preparation of the manuscript?<br>• **NO** - Include this sentence at the end of your statement: *The funders had no role in study design, data collection and analysis, decision to publish, or preparation of the manuscript.*<br>• **YES** - Specify the role(s) played.<br><br>* typeset	
**Competing Interests**<br><br>Use the instructions below to enter a competing interest statement for this submission. On behalf of all authors, disclose any competing interests that could be perceived to bias this work—acknowledging all financial support and any other relevant financial or non-financial competing interests.<br><br>This statement **will appear in the published article** if the submission is accepted. Please make sure it is accurate. View published research articles from *PLOS ONE* for specific examples.	The authors have declared that no competing interests exist.

**NO authors have competing interests**<br><br>Enter: *The authors have declared that no competing interests exist.*<br><br>**Authors with competing interests**<br><br>Enter competing interest details beginning with this statement:<br><br>*I have read the journal's policy and the authors of this manuscript have the following competing interests: [insert competing interests here]*<br><br>* typeset	
**Ethics Statement**<br><br>Enter an ethics statement for this submission. This statement is required if the study involved:<br><br>• Human participants<br>• Human specimens or tissue<br>• Vertebrate animals or cephalopods<br>• Vertebrate embryos or tissues<br>• Field research<br><br>Write "N/A" if the submission does not require an ethics statement.<br><br>General guidance is provided below. Consult the submission guidelines for detailed instructions. **Make sure that all information entered here is included in the Methods section of the manuscript.**	The study was approved by the Research Ethics Committee of the Faculty of Dentistry, Alexandria University, Egypt (IRB 00010556)-(IORG 0008839). In an online survey, participants who completed the survey were considered to have provided written implicit consent.

Powered by Editorial Manager® and ProduXion Manager® from Aries Systems Corporation

**Format for specific study types**<br><br>**Human Subject Research (involving human participants and/or tissue)**<br>• Give the name of the institutional review board or ethics committee that approved the study<br>• Include the approval number and/or a statement indicating approval of this research<br>• Indicate the form of consent obtained (written/oral) or the reason that consent was not obtained (e.g. the data were analyzed anonymously)<br><br>**Animal Research (involving vertebrate animals, embryos or tissues)**<br>• Provide the name of the Institutional Animal Care and Use Committee (IACUC) or other relevant ethics board that reviewed the study protocol, and indicate whether they approved this research or granted a formal waiver of ethical approval<br>• Include an approval number if one was obtained<br>• If the study involved *non-human primates*, add *additional details* about animal welfare and steps taken to ameliorate suffering<br>• If anesthesia, euthanasia, or any kind of animal sacrifice is part of the study, include briefly which substances and/or methods were applied<br><br>**Field Research**<br><br>Include the following details if this study involves the collection of plant, animal, or other materials from a natural setting:<br>• Field permit number<br>• Name of the institution or relevant body that granted permission	
**Data Availability**<br><br>Authors are required to make all data underlying the findings described fully available, without restriction, and from the time of publication. PLOS allows rare exceptions to address legal and ethical concerns. See the PLOS Data Policy and FAQ for detailed information.	Yes - all data are fully available without restriction

A Data Availability Statement describing where the data can be found is required at submission. Your answers to this question constitute the Data Availability Statement and **will be published in the article**, if accepted.<br><br>**Important:** Stating 'data available on request from the author' is not sufficient. If your data are only available upon request, select 'No' for the first question and explain your exceptional situation in the text box.<br><br>Do the authors confirm that all data underlying the findings described in their manuscript are fully available without restriction?	
**Describe where the data may be found in full sentences. If you are copying our sample text, replace any instances of XXX with the appropriate details.**<br><br>• If the data are **held or will be held in a public repository**, include URLs, accession numbers or DOIs. If this information will only be available after acceptance, indicate this by ticking the box below. For example: *All XXX files are available from the XXX database (accession number(s) XXX, XXX.*).<br>• If the data are all contained **within the manuscript and/or Supporting Information files**, enter the following: *All relevant data are within the manuscript and its Supporting Information files.*<br>• If neither of these applies but you are able to provide **details of access elsewhere**, with or without limitations, please do so. For example:<br><br>*Data cannot be shared publicly because of [XXX]. Data are available from the XXX Institutional Data Access / Ethics Committee (contact via XXX) for researchers who meet the criteria for access to confidential data.*<br><br>*The data underlying the results presented in the study are available from (include the name of the third party*	The dataset is attached as an Excel file.

and contact information or URL).<br>• This text is appropriate if the data are owned by a third party and authors do not have permission to share the data.<br><br>* typeset	
Additional data availability information:	

Powered by Editorial Manager® and ProduXion Manager® from Aries Systems Corporation

Manuscript

Click here to access/download;Manuscript;Manuscript stress 170720.docx

Behavior change due to COVID-19 among dental academics - The theory of planned behavior: stresses, worries, training, and pandemic severity.

Nour Ammar[1], Nourhan M. Aly[1], Morenike O. Folayan[2], Yousef Khader[3], Jorma Virtanen[4], Ola B. Al-Batayneh[5], Simin Z. Mohebbi[6], Sameh Attia[7], Hans-Peter Howaldt[7], Sebastian Boettger[7], Diah A. Maharani[8], Anton Rahardjo[8], Imran Khan[9], Marwa Madi[10], Maher Rashwan[11], Verica Pavlic[12], Smiljka Cicmil[12], Youn-Hee Choi[13], Easter Joury[14], Jorge L. Castillo[15], Kanako Noritake[16], Anas Shamala[17], Gabriella Galluccio[18], Antonella Polimeni[18], Prathip Phantumvanit[19], Davide Mancino[20], Jin-Bom Kim[21], Maha M. Abdelsalam[22], Arheiam Arheiam[23], Mai A. Dama[24], Myat Nyan[25], Iyad Hussein[26] , Mohammad M. Alkeshan[27], Ana P. Vukovic[28], Alfredo Iandolo[29], Arthur M. Kemoli[30], Maha El Tantawi[1*].

[1] Department of Pediatric Dentistry and Dental Public Health, Faculty of Dentistry, Alexandria University, Alexandria, Egypt.

[2] Department of Child Dental Health, Obafemi Awolowo University, Ile-Ife, Nigeria.

[3] Department of Public Health, Jordan University of Science and Technology, Irbid, Jordan.

[4] Department of Clinical Dentistry, Faculty of Medicine, University of Bergen, Bergen, Norway.

[5] Department of Preventive Dentistry, Faculty of Dentistry, Jordan University of Science and Technology, Irbid, Jordan.

[6] Research Center for Caries Prevention, Dentistry Research Institute, Tehran University of Medical Sciences, Tehran, Iran. Professor, Community Oral Health Department, School of Dentistry, Tehran University of Medical Sciences, Tehran, Iran.

[7] Department of Cranio-Maxillofacial Surgery, Justus-Liebig University Giessen, Giessen, Germany.

[8] Department of Preventive and Public Health Dentistry, Faculty of Dentistry, Universitas Indonesia, Depok, Indonesia.

[9] Department of Oral & Maxillofacial Surgery, Faculty of Dentistry, Jamia Millia Islamia, New Delhi, India.

[10] Department of Preventive Dental Sciences, College of Dentistry, Imam Abdulrahman Bin Faisal University, Dammam, Saudi Arabia.

[11] Center for Oral Bioengineering, Barts and the London, School of Medicine and Dentistry, Queen Mary University of London, London, UK, and an assistant lecturer, Department of Conservative Dentistry, Faculty of Dentistry, Alexandria University, Alexandria, Egypt.

[12] Department of Oral Rehabilitation, Faculty of Medicine Foca, University of East Sarajevo, Bosnia and Herzegovina.

[13] Department of Preventive Dentistry, School of Dentistry, Kyungpook National University, Republic of Korea.

[14] Centre for Dental Public Health and Primary Care, Institute of Dentistry, Barts and The London School of Medicine and Dentistry, Queen Mary University of London, London, United Kingdom.

[15] Department of Dentistry for Children and Adolescents, Universidad Peruana Cayetano Heredia, Lima, Peru.

[16] Oral Diagnosis and General Dentistry department, Dental Hospital, Tokyo Medical and Dental University, Tokyo, Japan.

[17] Department of Biological & Preventive Sciences, College of Dentistry, University of Science & Technology, Sanaa, Yemen.

[18] Department of Oral and Maxillo Facial Sciences, Faculty of Medicine and Dentistry, Sapienza University of Rome, Rome, Italy.

[19] Faculty of Dentistry, Thammasat University, Bangkok, Thailand.

[20] Department of Endodontics and Conservative Dentistry, Faculty of Dental Medicine, University of Strasbourg, Strasbourg 67000, France, and a professor, Department of Biomaterials and Bioengineering, INSERM UMR_S 1121, Strasbourg University, Strasbourg 67000, France.

[21] Department of Preventive and Community Dentistry, School of Dentistry, Pusan National University, Republic of Korea.

[22] Department of Biomedical Dental Sciences, College of Dentistry, Imam Abdulrahman Bin Faisal University, Dammam, Saudi Arabia.

[23] Department of Community and Preventive Dentistry, Faculty of Dentistry, University of Benghazi, Libya.

[24] Orthodontics and Pediatric Dentistry Department, Faculty of Dentistry, Arab American University, Palestine.

[25] Department of Prosthodontics, University of Dental Medicine, Mandalay, Myanmar.

[26] Department of Pediatric Dentistry, Mohammed Bin Rashid University of Medicine and Health Sciences, Dubai, United Arab Emirates.

[27] Department of Pediatric Dentistry, Seoul National University Dental Hospital, Seoul, South Korea.

[28] Department of Pediatric and Preventive Dentistry, School of Dental Medicine, University of Belgrade, Belgrade, Serbia.

[29] Department of Endodontics, University of Salerno, Fisciano, Italy.

[30] Department of Paediatric Dentistry & Orthodontics, School of Dental Sciences, University of Nairobi, Kenya.

*Corresponding author:

Email: maha_tantawy@hotmail.com (MET)

Abstract

Objective

COVID-19 pandemic led to major life changes. We assessed the psychological impact of COVID-19 on dental academics globally and on changes in their behaviors.

Method

We invited dental academics to complete a cross-sectional, online survey from March to May 2020. The survey was based on the Theory of Planned Behavior (TPB). The survey collected data on participants' stress levels (using the Impact of Event Scale), attitude (fears, and worries because of COVID-19 extracted by Principal Component Analysis (PCA)), perceived control (resulting from training on public health emergencies), norms (country-level COVID-19 fatality rate), and personal and professional backgrounds. We used multilevel regression models to assess the association between the study outcome variables (frequent handwashing and avoidance of crowded places) and explanatory variables (stress, attitude, perceived control and norms).

Results

1862 academics from 28 countries participated in the survey (response rate= 11.3%). Of those, 53.4% were female, 32.9% were <46 years old and 9.9% had severe stress. PCA extracted three main factors: fear of infection, worries because of professional responsibilities, and because of restricted mobility. These factors had significant dose-dependent association with stress and were significantly associated with more frequent handwashing (B= 0.56, 0.33, and 0.34) and avoiding crowded places (B= 0.55, 0.30, and 0.28). Low country fatality rates were significantly associated with more handwashing (B= -2.82) and avoiding crowded places (B= -6.61). Training was not significantly associated with behavior change (B= -0.01 and -0.11).

Conclusions

COVID-19 had a considerable psychologic impact on dental academics. There was a direct, dose-dependent association between change in behaviors and worries but no association with training. More change in behaviors was associated with lower country COVID-19 fatality rates. Fears and stresses were associated with greater adoption of preventive measures against the pandemic.

Keywords

Dental faculty; COVID-19; psychological stresses; fear; training; surveys and questionnaires; multilevel analysis

Introduction

The novel coronavirus (COVID-19) pandemic has influenced all life aspects. The highly contagious nature of the disease and its fatal outcomes led to changes in lifestyle for many people. (1) These lifestyle changes included social distancing, avoiding public places, more frequent hand washing, and wearing face masks in public. (2) These changes were sometimes associated with stress-inducing factors such as temporary unemployment, working from home, home-schooling of children, lack of physical contact with other family members, friends, and colleagues, and worrying that loved ones and important others may be infected. (3,4)

Researchers and academics also face the psychological impact of the COVID-19 pandemic. The sudden closure of schools mandated the adoption of e-learning technologies. This, coupled with the suspension of several research projects (5) and unemployment threats (6) may have created new stresses and added to already existing mental health conditions associated with work-life conflict. (7,8)

Healthcare workers are at greater risk of COVID-19 infection than the general population because of their frequent contact with affected individuals. Dental professionals are especially vulnerable to infections during pandemics. (8) Dental academics – educators who train dental students – face high levels of stress resulting from heavy work overload in addition to anxiety and fear attributed to their greater risk of infection during treatment provision in the dental office, especially during pandemics. (9,10) Mild anxiety during pandemics is natural and may foster preventive behaviors. (11) However, severe persistent anxiety and fear are powerful emotions that affect both physical and mental well-being. (12,13)

The theory of planned behavior (TPB) posits that behaviors can be predicted by intentions to engage in these behaviors. (14) These intentions, in turn, are affected by the control that people perceive they have over their actions, by their attitude toward the behavior

and whether they think it is useful, important or desirable, and by the norms they perceive to be prevailing around them. The TPB was previously used to explain dentists' behaviors including delivering prevention, (15) reporting suspected violence, (16) and managing drug users. (17) The change in behaviors among dentists due to the COVID-19 pandemic may be explained by the TPB including the control they perceive they have over avoiding infection by the disease because of previous training they received, worries because of the pandemic which may affect their attitudes and the importance they attach to adopting preventive behaviors, and the prevailing norms around them regarding the seriousness of the pandemic based on the fatalities it causes. Adopting preventive measures to avoid infection protects health care professionals, their families, patients, and the public. It is important to understand the factors associated with these behaviors and if they are impaired by the levels of stress the professionals have.

This study aimed to assess the psychological impact of the COVID-19 pandemic on dental academics and on changes in their behaviors as a result of the pandemic in several countries. The hypothesis of the study was that the TPB components are associated with change in dental academics' behaviors due to the pandemic.

Methods

Design

This was a cross-sectional study that used an online, multi-country survey to collect data from dental academics in several countries around the world between March and May 2020. Ethical approvals for the study were obtained from Alexandria University, Egypt (IRB 00010556)-(IORG 0008839)/6-11-2016) and other institutions in participating countries.

Participants and sampling

The study participants were a convenience sample of dental academics identified through emails posted on institutional websites in addition to direct invitation from country collaborators who reached them through professional social media groups or email lists. Participants were invited if they were dental educators training students in universities or institutions at the time of the study regardless of their degree (BDS, master or higher) or title (professors, associate or assistant professors or less) including clinical instructors and if they consented to participate.

Countries from which participants were recruited are listed in Appendix 1. Sample size was based on assuming a 95% confidence level, 5% margin of error, and prevalence of severe stress=10%. (18,19) The calculated number of participants also ensured adequate power for Principal Component Analysis (PCA) which requires at least 100 participants. (20)

Study questionnaire

An anonymous, close-ended questionnaire was developed for the study. The questionnaire consisted of four sections; section 1 included the 15-item Impact of Event Scale (IES) (21,22) which assessed post-traumatic responses to certain events- in this case, COVID-19. Its internal consistency and validity were previously demonstrated. (21) In the present study, its Cronbach alpha was 0.83 indicating high internal consistency. Items were scored on a 4-point Likert scale; 0= not at all, 1= rarely, 3= sometimes, and 5= often. Adding the scores of all items gave the total score which was categorized into subclinical, mild stress, moderate stress, and severe stress using cutoff points of 0-8, 9-25, 26-43, and 44+. (22) Section 2 included 16 items assessing participants' attitudes toward the impact of the COVID-19. Participants indicated how much these items caused them worry on a scale from 1 (not worried at all) to 10 (extremely worried). Section 3 assessed participants' agreement

with two statements describing change in behavior because of the COVID-19 pandemic (frequent handwashing and avoiding crowded places) on a scale from 1 (strongly disagree) to 10 (strongly agree). Section 4 was a 9 item close-ended questionnaire about participants' personal and professional background including sex, age, country, living arrangements, highest academic degree obtained, whether the participants coordinate courses, have clinical responsibilities, hold administrative positions, and whether they received training on public health emergencies (Appendix 2).

The questionnaire was uploaded to SurveyMonkey. Participants were asked to select only one response per question and they were allowed to make one submission. No IPs or emails were collected to ensure confidentiality. The questionnaire was preceded by a brief introduction explaining the purpose of the study, assuring participants of the confidentiality of their responses, and emphasizing that their participation was voluntary. After SurveyMonkey settings were modified, the survey was tested for face and content validity by five academics who were not involved in the study to ensure clarity and relevance of the questionnaire. The questionnaire was developed in English. In addition, two versions were prepared for use in Iran and Brazil where it was translated by collaborators/ dentists into Farsi and Portuguese followed by back translation to ensure accuracy.

Data collection

Survey links were sent to collaborators for distribution to participants who received the links on their emails or social media groups. Reminders were sent two weeks after the first invitation email to encourage participation.

Analysis

After the survey closure, the Excel sheets were downloaded, cleaned, and imported to SPSS version 23.0 for analysis (IBM Corp., Armonk, N.Y., USA). Frequencies, percentages, means, and standard deviations were calculated for descriptive statistics.

Prior to PCA, the suitability of data for this analysis was assessed. The Kaiser-Meyer-Olkin (KMO) measure of sampling adequacy was 0.91 which is above the recommended value of 0.6. The P-value of Bartlett's test of Sphericity (23) was statistically significant ($P < 0.0001$), supporting the use of PCA. Major attitude components were, therefore, extracted from the 16 items in section 2 of the survey. Extraction was based on eigenvalues >1. Varimax rotation with Kaiser normalization was used and loading coefficients < 0.4 were suppressed to facilitate interpretation of factor loading. Regression coefficients of the factors extracted from the PCA were saved to the dataset and used as explanatory variables for further analysis.

Two types of outcomes were assessed. The first was the stress levels based on the categories of IES. These were included in a multilevel ordinal logistic regression where the explanatory variables were the major worries/ attitudes derived from the PCA, the ratio between the number of COVID-19 deaths to cases per million at country level (fatality rate) (24) and whether the participant received training to manage public health emergencies. The model controlled for confounders (personal and professional background factors) which were introduced as fixed effects and country was included in the model as a random effect factor. The second set of outcomes was the scores indicating change in behaviors due to the COVID-19 pandemic (frequent handwashing and avoiding crowded places). These were included in two multilevel linear regression models with the same explanatory variables representing the TPB components (major attitude/ worries, fatality rate at country level, and receiving training) in addition to stress levels. These models also controlled for the confounders

(personal and professional background variables) and included country as a random effect factor. Regression coefficients (and odds ratios for the ordinal logistic regression model) and 95% confidence intervals were calculated. Significance was set at 5%.

Results

Responses were received from 1862 participants from 28 countries with an overall response rate= 11.3%. Almost half the participants (53.8%) were from Iran, USA, India, Germany and, Indonesia (Appendix 1). About 53.4% were females, 32.9% were >35-45 years old, 66.3% were living with partner/ spouse, 48.7% were PhD degree holders, 85.6% coordinated courses, 87.4% had clinical responsibilities, 52.9% had administrative positions and 51.7% had not received training for public health emergencies. The mean (SD) fatality rate at country-level as of May 25th, 2020 was 0.06 (0.04) (data not shown in the table). Also, 9.9% had severe stress, 37.5% had moderate stress and 39.6% had mild stress (Table 1).

Table 1: Personal and professional background of dental academics and their levels of COVID-19- related stress (n= 1862)

Factors		N (%)
Sex	Male	869 (46.6)
	Female	996 (53.4)
Age in years	25-35	519 (27.8)
	>35-45	614 (32.9)
	>45-55	376 (20.2)
	>55- 65	256 (13.7)
	>65	100 (5.4)

Living arrangements	Alone	198 (10.6)
	With parents	281 (15.1)
	With partner/ spouse	1236 (66.3)
	Shared accommodation	68 (3.6)
	Other	82 (4.4)
Highest academic degree obtained	BDS	337 (18.1)
	MSc	619 (33.2)
	PhD	909 (48.7)
Coordinates courses	No	269 (14.4)
	Yes	1596 (85.6)
Has clinical responsibilities	No	235 (12.6)
	Yes	1630 (87.4)
Has administrative position	No	878 (47.1)
	Yes	987 (52.9)
Received training for public health emergencies	No	964 (51.7)
	Yes	901 (48.3)
Stress levels	Subclinical	242 (13.0)
	Mild	739 (39.6)
	Moderate	700 (37.5)
	Severe	184 (9.9)

Table 2 highlights the PCA and factor loadings for major worries and attitudes related to the COVID-19 pandemic. Three components explaining 67.3% of the variance were extracted by PCA from the 16 items with factor loadings ranging from 0.735 to 0.823. In the first component, seven items had loadings ≥ 0.735 and were related to fear of infection. In the

second component, five items had loadings ≥ 0.737 and were related to worries from professional responsibilities. The last component included four items with factor loadings ≥ 0.754 and it was about worries from restricted mobility. The greatest fear of infection was that important others would get COVID-19 infection because of the participant (mean= 7.66). The greatest worry about professional responsibilities was related to the required material during the pandemic (mean= 6.48). The greatest worry because of restricted mobility was caused by restricted mobility within the country (mean= 6.70).

Table 2: Principal Component Analysis and factor loadings for major worries and attitudes related to COVID-19 pandemic

		Factor loadings		
	Mean (SD)	Fear of infection	Professional responsibilities	Restricted mobility
Catching COVID-19 infection from a colleague	5.83 (2.74)	0.768		
Catching COVID-19 infection from a patient	7.20 (2.80)	0.786		
Catching COVID-19 infection from a student	5.61 (2.96)	0.777		
Catching COVID-19 infection from a source not related to work	6.41 (2.61)	0.735		
Important others getting infected with COVID-19 because of me	7.66 (2.76)	0.750		

Important others getting infected with COVID-19 because of another source	7.64 (2.48)	0.747		
Patients getting infected with COVID-19	7.15 (2.63)	0.755		
Finishing open courses satisfactorily because of the COVID-19 outbreak	6.18 (2.73)		0.774	
Teaching students required material because of the COVID-19 outbreak	6.48 (2.68)		0.823	
Supporting students psychologically during the COVID-19 outbreak	6.44 (2.67)		0.737	
Managing online learning during the COVID-19 outbreak	6.45 (2.75)		0.783	
Finishing required reports/ assignments during the COVID-19 outbreak	6.23 (2.76)		0.749	
Restricted mobility in my country because of the COVID-19 outbreak	6.70 (2.91)			0.769
Restricted mobility from/ to my country because of the COVID-19 outbreak	6.13 (3.24)			0.817
Restricted mobility affecting sports and social activities because of COVID-19	6.33 (2.85)			0.790

Missing events important to my career because of the COVID-19 outbreak	5.96 (2.94)		0.754

KMO= 0.91, P value of Bartlett’s test< 0.0001

Table 3 highlights the factors associated with stress levels among dental academics. Fear of infection, worries about professional responsibilities and restricted mobility, country-level fatality rate, and previous training on public health emergencies were significantly associated with severe, moderate, and mild stress (P< 0.0001). Fear of infection had a significant, direct, and dose-dependent association with stress with higher scores of fear associated in a gradient with mild (OR=1.186), moderate (OR=1.465), and severe stress (OR= 1.483).

Similarly, there was a significant, direct, and dose-dependent association between worries due to professional responsibilities and higher levels of stress: worries were associated in a gradient with mild (OR=1.209), moderate (OR=1.317) and severe stress (OR= 1.369). The direct, dose-dependent association between worries due to restricted mobility and stresses also followed a gradient with mild (OR=1.010), moderate (OR=1.302), and severe stress (OR= 1.379).

A stronger dose-dependent relationship was observed in the association between country-level fatality rate and severe (OR= 6.893), moderate (OR= 1.539), and mild stresses (OR= 0.947); higher fatality rate was associated with higher odds of severe and moderate stress but lower odds of mild stress. The association between stress levels and receiving training was U shaped; training was associated with higher odds of severe (OR= 1.040) and mild stress (OR= 1.084) and lower odds of moderate stress (OR= 0.971).

Table 3: Factors associated with stress levels among dental academics (n= 1862)

Stressors	OR (95% CI): vs subclinical stress		
	Severe	Moderate	Mild
Fear of infection	1.483 (1.481, 1.484)*	1.465 (1.464, 1.466)*	1.186 (1.184, 1.188)*
Worries about professional issues	1.369 (1.368, 1.369)*	1.317 (1.316, 1.318)*	1.209 (1.208, 1.210)*
Worries about restricted mobility	1.379 (1.378, 1.380)*	1.302 (1.300, 1.305)*	1.010 (1.008, 1.012)*
COVID-19 fatality rate	6.893 (6.891, 6.893)*	1.539 (1.539, 1.540)*	0.947 (0.935, 0.939)*
Received training vs not	1.040 (1.038, 1.042)*	0.973 (0.971, 0.974)*	1.084 (1.083, 1.086)*

Multilevel ordinal logistic regression controlling for sex, age, living arrangements, highest academic degree obtained, course coordination, having clinical responsibilities, having administrative positions as fixed factors and country included as a random factor; 48.7% correctly classified. OR: odds ratio, CI: confidence interval, *: statistically significant at P< 0.05

Table 4 shows the factors associated with behavior change as a result of the COVID-19 pandemic in multilevel linear regression analysis. Participants agreed that they avoided crowded places (mean= 8.14) and washed their hands frequently (mean= 8.06). Compared to subclinical stress, severe stress was significantly and directly associated with more frequent handwashing (B= 0.93) and avoiding crowded places (B= 0.62). Also, compared to subclinical stress, moderate and mild stresses were significantly associated with more

frequent handwashing (B= 0.83 and B= 0.67) but had no significant association with avoiding crowded places. The dose-dependent associations between stress severity and change in each behavior followed a gradient with greater changes reported by participants with higher levels of stress.

Greater fear of infection, worries about professional responsibilities and worries because of restricted mobility were associated with more frequent handwashing (B= 0.56, 0.33 and 0.34) and more avoidance of crowded places (B= 0.55, 0.30 and 0.28).

Higher COVID-19 fatality rates were associated with less frequent handwashing (B= -2.82) and less avoidance of crowded places (B= -6.61). The associations between receiving training and changes in the two behaviors were not statistically significant (P< 0.05).

Table 4: Association between change in behaviors due to COVID-19 and stresses, worries, COVID-19 fatality rate, and training among dental academics (n= 1862)

Factors	B (95% CI)	
	Frequent handwashing	Avoiding crowded places
Change in behavior scale: Mean (SD)	8.06 (2.40)	8.14 (2.41)
Severe vs subclinical stress	0.93 (0.46, 1.40)*	0.62 (0.15, 1.10)*
Moderate vs subclinical stress	0.83 (0.48, 1.19)*	0.33 (-0.03, 0.68)
Mild vs subclinical stress	0.67 (0.33, 1.01)*	0.26 (-0.08, 0.60)
Fear of infection	0.56 (0.45, 0.67)*	0.55 (0.44, 0.66)*
Worries about professional responsibilities	0.33 (0.23, 0.44)*	0.30 (0.19, 0.40)*
Worries about restricted mobility	0.34 (0.24, 0.45)*	0.28 (0.18, 0.39)*
COVID-19 fatality rate	-2.82 (-5.32, -0.32)*	-6.61 (-9.13, -4.08)*

Received training vs not	-0.01 (-0.21, 0.20)	-0.11 (-0.32, 0.10)

Multilevel linear regression controlling for sex, age, living arrangements, highest academic degree obtained, course coordination, clinical responsibilities, having administrative positions as fixed factors and country as random factor, B: regression coefficient, CI: confidence interval, *: statistically significant at $P < 0.05$.

Discussion

The findings indicated that the COVID-19 pandemic was a stress inducer for dental academics, with approximately 10% having severe COVID-19-related traumatic stress. The main sources of stress were fear of contracting infection, restricted mobility due to the lockdown enforced in most countries to control the spread of the pandemic, and worries because of professional responsibilities related to teaching and research. Measures taken by individuals to contain the infection included avoidance of crowded places, and washing hands more frequently. Training on public health emergencies was significantly associated with stresses but not with change in behaviors due to the pandemic. A dose-dependent relationship existed between severity of stresses and worries related to fear of infection, teaching and research responsibilities and restricted mobility. A direct, dose-dependent relationship also existed between stress levels and change in behaviors due to the pandemic. Dose-dependent associations were suggested by Hill among the criteria supporting causality in observational studies when clinical trials cannot be conducted. (25) However, dose-dependent associations are not proof of causality on their own and the most important criterion of causality; time sequence where exposure precedes outcome, can only be ascertained in a longitudinal study. The study hypothesis was, thus, partly supported: not all components of the TPB were significantly associated with change in behaviors due to COVID-19.

One of the strengths of the study was the diversity of countries represented by the study participants. This enabled the study to generate data representing different educational systems and backgrounds thereby increasing the generalizability of the findings. Also, the study used validated tools with high internal consistency and/ or factor loadings. In addition, the study captured the psychological impact of the pandemic at its early stages thus providing important and valuable information that can be used in designing support systems for dental academics.

The study, however, had some limitations. First, data were collected at different stages of the pandemic in various countries and this may have confounded the assessment of the level of stress. In addition, the study was cross-sectional and thus, cannot prove causality. Also, the response rate was low similar to previous research (26) and this may be attributed to the psychological impact of the pandemic with resulting possible underestimation of the level of stress reported in the study since those with higher levels of stress may be more likely to ignore the survey. Despite these limitations, the study highlights the psychological impact of the pandemic on dental educators who are critical stakeholders in the education and healthcare sectors. As countries pass through the first wave of the pandemic, more attention will need to be paid to the psychological impact of the pandemic on people's lives because of its possible effect on productivity, wellbeing, health, and quality of life. (27)

We found a direct association between fears and worries and behavior changes in agreement with previous studies including British adults (28), a nationally representative sample of Americans (29) and lay persons from ten countries in Europe, America and Asia. (30) These studies reported an association between perceived risk of infection, fatality risk or negative emotions such as fear and anxiety and greater adoption of COVID-19 preventive behaviors such as hand hygiene and social distancing. Our findings and those of other studies

suggest that fear may trigger a protective reaction through the adoption of preventive measures to reduce risks.

Lower COVID-19 fatality rates were observed in countries where dental academics reported more frequent handwashing and more avoidance of crowded places. Dental academics' COVID-19-related behaviors reflect the behaviors of the general populations. Risk reduction communication undertaken as part of the public health response might have led to the adoption of COVID-19 preventive measures resulting in lower rates of COVID-19 spread and fatality. However, the cross-sectional design of the study does not show time sequence and the direction of the relationship between fatality rates and adoption of preventive measures cannot be elucidated. Future longitudinal studies are needed to establish cause and effect and allow the disentanglement of these complex associations.

The present study showed a 10% prevalence of severe COVID-19-induced stress among dental academics; a higher level than the 7% reported among the general public in Wuhan, China (18) and 8.7% general anxiety reported among Italian dentists (19) and similar to the 11.5% among Israeli dentists and dental hygienists. (31) This indicates a need to provide support for dental academics' mental health. In addition, fear and anxiety among the educators may have a detrimental effect on dental students with long-lasting consequences on the profession. Few universities have instituted mental health support programs for their staff and students to cope with stresses even before the pandemic crisis. (32)

In the present study, COVID-19 was associated with severe stresses because of restricted mobility caused by isolation and quarantine, (33) as observed in past epidemics like the Ebola (34) and MERS. (35) The fear of transmitting infections to important others and loved ones was another COVID-19-related stress inducing factor observed in the present study similar to that reported by dentists from 30 countries in a previous study, (4) and it had a dose-dependent association similar to what was observed among Israeli dentists. (31)

At the present time when the pandemic spreads and death toll rises, it was hoped that training would prepare dental academics to adopt preventive measures. Our results, however, showed no significant effect of previous training on changing behaviors. Training on public health emergencies was associated with less stress up to a certain level beyond which the higher awareness of risks brought about by training was associated with more rather than less stress. Thus, whether in relation to change in behavior or reducing stresses, training was not associated with greater perceived control. This may be attributed to the generic nature training the academics received which did not address the specific needs related to COVID-19 prevention. This implies that appropriate responses to COVID-19 will require specific and tailored training different from the standard training for public health emergencies.

Conclusion

The present study showed a considerable psychologic impact of the COVID-19 pandemic on dental academics that was directly associated with fear of infection and worries because of professional responsibilities and restricted mobility. Changes in behaviors due to the pandemic and greater adoption of preventive measures were associated with stresses and worries in a direct and dose-dependent relationship but were not associated with training. Greater adoption of preventive measures was inversely related to COVID-19 fatality rates at country level.

Acknowledgments

We are grateful to all the academics who kindly responded to the survey and answered our questions in these difficult times.

References

1. Gralinski LE, Menachery VD. Return of the coronavirus: 2019-nCoV. Viruses. 2020;12(2).

2. World Health Organization. Coronavirus disease (COVID-19) advice for the public [Internet]. 2020 [cited 2020 Jun 20]. Available from: https://www.who.int/emergencies/diseases/novel-coronavirus-2019/advice-for-public

3. World Health Organization. Mental health and COVID-19 [Internet]. 2020 [cited 2020 Jun 20]. Available from: https://www.who.int/teams/mental-health-and-substance-use/covid-19

4. Ahmed MA, Jouhar R, Ahmed N, Adnan S, Aftab M, Zafar MS, et al. Fear and Practice Modifications among Dentists to Combat Novel Coronavirus Disease (COVID-19) Outbreak. Int J Environ Res Public Health [Internet]. 2020 Apr 19 [cited 2020 Jun 20];17(8):2821. Available from: https://www.mdpi.com/1660-4601/17/8/2821

5. The United Nations Educational Scientific and Cultural Organization, Caribbean II for HE in LA and the. COVID-19 and higher education: Today and tomorrow [Internet]. 2020 [cited 2020 Jun 20]. Available from: http://www.iesalc.unesco.org/en/wp-content/uploads/2020/04/COVID-19-EN-090420-2.pdf

6. McKibbin WJ, Fernando R. The Global Macroeconomic Impacts of COVID-19: Seven Scenarios. SSRN Electron J [Internet]. 2020 Mar 5 [cited 2020 Jun 20]; Available from: https://papers.ssrn.com/abstract=3547729

7. Gail Kinman, Siobhan Wray. Higher stress A SURVEY OF STRESS AND WELL-BEING AMONG STAFF IN HIGHER EDUCATION [Internet]. 2013 Jul [cited 2020 Jun 20]. Available from: www.ucu.org.uk

8. Meng L, Hua F, Bian Z. Coronavirus Disease 2019 (COVID-19): Emerging and Future

Challenges for Dental and Oral Medicine. J Dent Res. 2020 May 1;99(5):481–7.

9. Rutter H, Herzberg J, Paice E. Stress in doctors and dentists who teach. Med Educ [Internet]. 2002 Jun 1 [cited 2020 Jun 20];36(6):543–9. Available from: http://doi.wiley.com/10.1046/j.1365-2923.2002.01229.x

10. Pereira LJ, Pereira CV, Murata RM, Pardi V, Pereira-Dourado SM. Biological and social aspects of Coronavirus Disease 2019 (COVID-19) related to oral health. Braz Oral Res. 2020;34.

11. Vinkers CH, van Amelsvoort T, Bisson JI, Branchi I, Cryan JF, Domschke K, et al. Stress resilience during the coronavirus pandemic. Eur Neuropsychopharmacol. 2020 Jun 1;35:12–6.

12. Balon R. Mood, anxiety, and physical illness: Body and mind, or mind and body? Depress Anxiety. 2006;23(6):377–87.

13. Vindegaard N, Eriksen Benros M. COVID-19 pandemic and mental health consequences: Systematic review of the current evidence. Brain Behav Immun. 2020;

14. Ajzen I. The theory of planned behavior. Organ Behav Hum Decis Process. 1991 Dec 1;50(2):179–211.

15. Yusuf H, Kolliakou A, Ntouva A, Murphy M, Newton T, Tsakos G, et al. Predictors of dentists' behaviours in delivering prevention in primary dental care in England: using the theory of planned behaviour. BMC Health Serv Res. 2015;16(1):1–7.

16. El Tantawi M, Gaffar B, Arheiam A, Abdelaziz W, Al-Batayneh OB, Alhoti MF, et al. Dentists' intention to report suspected violence: A cross-sectional study in eight Arab countries. BMJ Open [Internet]. 2018 Mar 1 [cited 2020 Jun 21];8(3). Available from: https://pubmed.ncbi.nlm.nih.gov/29602845/

17. El Tantawi M, AlJameel ABH, Fita S, AlSahan B, Alsuwaiyan F, El Meligy O. Dentists' intentions to manage drug users: Role of theory of planned behaviour and

continuing education. Eur J Dent Educ. 2019 Aug 1;23(3):364–72.

18. Liu N, Zhang F, Wei C, Jia Y, Shang Z, Sun L, et al. Prevalence and predictors of PTSS during COVID-19 outbreak in China hardest-hit areas: Gender differences matter. Psychiatry Res. 2020 May 1;287:112921.

19. Consolo U, Bellini P, Bencivenni D, Iani C, Checchi V. Epidemiological Aspects and Psychological Reactions to COVID-19 of Dental Practitioners in the Northern Italy Districts of Modena and Reggio Emilia. Int J Environ Res Public Health [Internet]. 2020 May 15 [cited 2020 Jun 20];17(10):3459. Available from: https://www.mdpi.com/1660-4601/17/10/3459

20. Abdi H, Williams LJ. Principal component analysis. Wiley Interdiscip Rev Comput Stat. 2010;2(4):433–59.

21. Horowitz M, Wilner N, Alvarez W. Impact of event scale: A measure of subjective stress. Psychosom Med. 1979;41(3):209–18.

22. Kolokotroni F. Impact of Event Scale BT - Encyclopedia of Quality of Life and Well-Being Research. In: Michalos AC, editor. Dordrecht: Springer Netherlands; 2014. p. 3102–5. Available from: https://doi.org/10.1007/978-94-007-0753-5_1377

23. Bartlett MS. A note of the multiplying factors for various chi square approximations. J R Stat Soc. 1954;Series B(16):296–298.

24. Worldometer. Coronavirus Cases. Worldometer. 2020. p. 1–22.

25. HILL AB. THE ENVIRONMENT AND DISEASE: ASSOCIATION OR CAUSATION? Proc R Soc Med [Internet]. 1965 May;58(5):295–300. Available from: https://pubmed.ncbi.nlm.nih.gov/14283879

26. Funkhouser E, Vellala K, Baltuck C, Cacciato R, Durand E, McEdward D, et al. Survey Methods to Optimize Response Rate in the National Dental Practice–Based Research Network. Eval Heal Prof [Internet]. 2017 Sep 1 [cited 2020 Jun

21];40(3):332–58. Available from: https://pubmed.ncbi.nlm.nih.gov/26755526/

27. Horesh D, Brown AD. Covid-19 response: Traumatic stress in the age of Covid-19: A call to close critical gaps and adapt to new realities. Psychol Trauma Theory, Res Pract Policy [Internet]. 2020 [cited 2020 Jun 20];12(4):331–5. Available from: https://pubmed.ncbi.nlm.nih.gov/32271070/

28. Harper CA, Satchell LP, Fido D, Latzman RD. Functional Fear Predicts Public Health Compliance in the COVID-19 Pandemic. Int J Ment Health Addict [Internet]. 2020 [cited 2020 Jun 20];1. Available from: /pmc/articles/PMC7185265/?report=abstract

29. de Bruin WB, Bennett D. Relationships Between Initial COVID-19 Risk Perceptions and Protective Health Behaviors: A National Survey. Am J Prev Med [Internet]. 2020 May [cited 2020 Jun 20];0(0). Available from: http://www.ajpmonline.org/article/S0749379720302130/fulltext

30. Dryhurst S, Schneider CR, Kerr J, Freeman ALJ, Recchia G, van der Bles AM, et al. Risk perceptions of COVID-19 around the world. J Risk Res [Internet]. 2020 May 5 [cited 2020 Jun 20];1–13. Available from: https://www.tandfonline.com/doi/full/10.1080/13669877.2020.1758193

31. Shacham M, Hamama-Raz Y, Kolerman R, Mijiritsky O, Ben-Ezra M, Mijiritsky E. COVID-19 Factors and Psychological Factors Associated with Elevated Psychological Distress among Dentists and Dental Hygienists in Israel. Int J Environ Res Public Health [Internet]. 2020 Apr 22 [cited 2020 Jun 20];17(8):2900. Available from: https://www.mdpi.com/1660-4601/17/8/2900

32. Fernandez A, Howse E, Rubio-Valera M, Thorncraft K, Noone J, Luu X, et al. Setting-based interventions to promote mental health at the university: a systematic review. Int J Public Health. 2016;61(7):797–807.

33. Brooks SK, Webster RK, Smith LE, Woodland L, Wessely S, Greenberg N, et al. The

psychological impact of quarantine and how to reduce it: rapid review of the evidence. Lancet [Internet]. 2020 Mar 14 [cited 2020 Jun 20];395(10227):912–20. Available from: /pmc/articles/PMC7158942/?report=abstract

34. Caleo G, Duncombe J, Jephcott F, Lokuge K, Mills C, Looijen E, et al. The factors affecting household transmission dynamics and community compliance with Ebola control measures: A mixed-methods study in a rural village in Sierra Leone. BMC Public Health [Internet]. 2018 Feb 13 [cited 2020 Jun 20];18(1). Available from: /pmc/articles/PMC5812186/?report=abstract

35. Jeong H, Yim HW, Song YJ, Ki M, Min JA, Cho J, et al. Mental health status of people isolated due to Middle East Respiratory Syndrome. Epidemiol Health [Internet]. 2016 [cited 2020 Jun 20];38:e2016048. Available from: https://pubmed.ncbi.nlm.nih.gov/28196409/

Supporting information

Appendix 1 (This is the 'S1 Appendix 1.pdf' file): Countries participating in the study and number of participants

Appendix 2 (This is the 'S2 Appendix 2.pdf' file): Survey for dental academics' stresses at the time of the COVID-19 outbreak

Appendix 1

Click here to access/download
Supporting Information
S1 Appendix 1.pdf

Appendix 2

Click here to access/download
Supporting Information
S2 Appendix 2.pdf

Dataset

Click here to access/download
Supporting Information
Dataset.xlsx

Related submission

Click here to access/download
Supporting Information
Related submission.docx
